# Directly-observed therapy (DOT) for the radical 14-day primaquine treatment of *Plasmodium vivax *malaria on the Thai-Myanmar border

**DOI:** 10.1186/1475-2875-9-308

**Published:** 2010-11-01

**Authors:** Rie Takeuchi, Saranath Lawpoolsri, Mallika Imwong, Jun Kobayashi, Jaranit Kaewkungwal, Sasithon Pukrittayakamee, Supalap Puangsa-art, Nipon Thanyavanich, Wanchai Maneeboonyang, Nicholas PJ Day, Pratap Singhasivanon

**Affiliations:** 1 Department of Tropical Hygiene, Faculty of Tropical Medicine, Mahidol University, Bangkok, Thailand; 2 Department of Clinical Tropical Medicine, Faculty of Tropical Medicine, Mahidol University, Bangkok, Thailand; 3 Department of International Medical Cooperation, National Center for Global Health and Medicine, Tokyo, Japan; 4 Mahidol-Oxford Tropical Medicine Research Unit (MORU), Faculty of Tropical Medicine, Mahidol University, Bangkok, Thailand

## Abstract

**Background:**

*Plasmodium vivax *has a dormant hepatic stage, called the hypnozoite, which can cause relapse months after the initial attack. For 50 years, primaquine has been used as a hypnozoitocide to radically cure *P. vivax *infection, but major concerns remain regarding the side-effects of the drug and adherence to the 14-day regimen. This study examined the effectiveness of using the directly-observed therapy (DOT) method for the radical treatment of *P. vivax *malaria infection, to prevent reappearance of the parasite within the 90-day follow-up period. Other potential risk factors for the reappearance of *P. vivax *were also explored.

**Methods:**

A randomized trial was conducted from May 2007 to January 2009 in a low malaria transmission area along the Thai-Myanmar border. Patients aged ≥ 3 years diagnosed with *P. vivax *by microscopy, were recruited. All patients were treated with the national standard regimen of chloroquine for three days followed by primaquine for 14 days. Patients were randomized to receive DOT or self-administered therapy (SAT). All patients were followed for three months to check for any reappearance of *P. vivax*.

**Results:**

Of the 216 patients enrolled, 109 were randomized to DOT and 107 to SAT. All patients recovered without serious adverse effects. The vivax reappearance rate was significantly lower in the DOT group than the SAT group (3.4/10,000 person-days vs. 13.5/10,000 person-days, *p *= 0.021). Factors related to the reappearance of vivax malaria included inadequate total primaquine dosage received (< 2.75 mg/kg), duration of fever ≤ 2 days before initiation of treatment, parasite count on admission ≥ 10,000/µl, multiple *P. vivax*-genotype infection, and presence of *P. falciparum *infection during the follow-up period.

**Conclusions:**

Adherence to the 14-day primaquine regimen is important for the radical cure of *P. vivax *malaria infection. Implementation of DOT reduces the reappearance rate of the parasite, and may subsequently decrease *P. vivax *transmission in the area.

## Background

Globally, over 3 billion people live in areas at risk of malaria infection; about one billion of these live in countries outside Africa, where malaria transmission is low and *Plasmodium vivax *is most prevalent [1,2]. Unlike *Plasmodium falciparum, P. vivax *infection rarely develops into complicated malaria and death is unusual. However, *P. vivax *has a dormant stage (the hypnozoite) that persists in the human liver and may cause relapse weeks, months, or even years later. Therefore, *P. vivax *infection is considered to have greater impact on morbidity than mortality, resulting in significant social and economic burdens [1]. Moreover, it is very difficult to control *P. vivax *transmission, because gametocytes appear almost simultaneously with schizonts.

In Thailand, most malaria cases are caused by *P. falciparum *and *P. vivax*. The incidence ratio of *P. falciparum *malaria to *P. vivax *malaria is almost 1:1 [3]. A three-day chloroquine regimen followed by 14 days of primaquine is the standard treatment regimen to eradicate *P. vivax *parasites in the bloodstream and hypnozoites in the liver. Although chloroquine resistance has been reported in Papua New Guinea and Indonesia [4], it is still effective in Thailand [5-8]. Occasional failure of the standard primaquine therapy (15 mg daily for 14 days) to prevent relapse has been observed [9-15]. However, primaquine resistance has not been confirmed. In Thailand, the relapse rates at day 28 are about 50% without primaquine therapy [16], and about 20% with standard primaquine therapy [17]. Relapse has not been observed among patients receiving high-dose primaquine therapy (30 mg daily for 14 days) [16].

A number of factors are reportedly associated with relapse, or the reappearance of *P. vivax*, including inadequate primaquine dosage [12,14], high parasitaemia at diagnosis, short duration of symptoms prior to diagnosis [14], presence of gametocytes on admission [18], age, and gender [6,14,19,20]. Because the radical cure of *P. vivax *hypnozoites requires 14 days of primaquine therapy, adherence to the drug regimen may greatly affect the prevention of relapse. Unfortunately, the effect of patient adherence on 14-day primaquine treatment, and its relation to preventing parasite reappearance, is not well-documented.

Directly-observed therapy (DOT) is an effective strategy to ensure patient adherence to long-term chemotherapy. Currently, DOT is the main strategy for treating tuberculosis (TB) in many countries and its success has been widely reported [21]. However, the effectiveness of DOT for 14-day primaquine therapy in vivax malaria has not been studied thoroughly, particularly in malaria-tuberculosis co-endemic countries. Here, a randomized control trial was conducted to determine the effectiveness of DOT for 14-day primaquine administration, and the reappearance of *P. vivax *in a low malaria transmission area along the Thai-Myanmar border. Other potential risk factors for the reappearance of *P. vivax *after radical treatment were also explored.

## Methods

### Study site

The study was conducted between May 2007 and January 2009 at the Rajanagarindra Tropical Disease International Centre (RTIC), Tanaosri Subdistrict, Suan Phung District, Ratchaburi Province, Thailand. The area is situated 100 km west of Bangkok near the Myanmar border. The study area comprised seven hamlets with about 3,500 residents. The population in the area is composed of four ethnic groups, Karen (85%), Thai (14%), Mon and Burmese (1%). For a decade, the RTIC clinic has provided free malaria diagnosis and treatment for the local community. Patients from other hamlets also routinely seek malaria treatment here.

### Study participants

Patients aged ≥ 3 years with microscopically-confirmed *P. vivax *infection were included in the study provided they or their guardians gave informed consent. Patients with microscopically confirmed mixed infections of *P. vivax *with other *Plasmodium *species, severe malaria, pregnant or lactating were excluded. G6PD deficient patients were included in this study, since the most common G6PD mutations in Thailand are G6PD Mahidol and G6PD Viangchan [22], which have been reported to be relatively safe for primaquine standard therapy [23]. This study was approved by the Ethics Committee of the Faculty of Tropical Medicine, Mahidol University.

### Randomization and treatment

To compare the effectiveness of directly observed therapy (DOT) with self-administered therapy (SAT), study participants were randomly assigned to either a DOT group or a SAT group using the block randomization method with a block size of four. Patients assigned to the SAT group, upon diagnosis, were given anti-malarials for self-administration with standard instructions in taking the drugs. Patients assigned to the DOT group were visited daily at home by RTIC staff until the full course was taken.

All patients were treated with the national standard regimen for vivax malaria, as recommended by the Malaria Control Program of Thailand (chloroquine 1,500 mg base over three days, followed by primaquine 15 mg daily for 14 days). G6PD screening is not required before giving the primaquine treatment. Chloroquine and primaquine doses were adjusted accordingly for patients under 14 years of age: for 8-13 years old, chloroquine 900 mg base over three days, and primaquine 10 mg daily for 14 days; for 3-7 years old, chloroquine 750 mg base over three days, and primaquine 5 mg daily for 14 days. If *P. vivax *parasitaemia reappeared within three months, the daily primaquine dose was increased to twice the initial dose.

### Data collection

Baseline demographic and clinical data were gathered by interview. Finger-prick blood samples were collected on filter paper to examine for G6PD mutations and parasite genotype. All patients were followed for 90 days with Day 0 defined as the first day of drug administration.

During the follow-up period, patients from both groups were visited by RTIC staff at Days 7, 14, 28, 60, and 90, to examine for *P. vivax *and other *Plasmodium *parasitaemia. Patients were instructed to visit RTIC if they experience any symptoms during the follow-up period. At Days 7 and 14, patients in the SAT group were asked whether they took their anti-malarials as instructed. Upon microscopic confirmation of *P. vivax *reappearance, a finger-prick blood sample was taken to determine the parasite genotype for comparison against baseline genotype(s).

### DNA extraction

DNA was extracted from finger-prick blood samples on filter paper, using a QIAamp DNA Mini Kit according to the manufacturer's instructions (Qiagen, Germany).

### G6PD mutation

The two most common G6PD mutations in the Karen population, G6PD Mahidol and G6PD Viangchan, were examined using polymerase chain reaction (PCR) and restriction fragment length polymorphism analysis of the PCR products (PCR-RFLP), as described by Nuchprayoon *et al *[24] with slight modification, as follows: the PCR reaction was carried out in a 20 µl reaction volume containing 2.5 mM MgCl_2_; for PCR-RFLP, 10 µl of the PCR product were digested with an appropriate restriction enzyme (New England Biolabs Inc., UK) in a total volume of 20 µl.

### Parasite genotyping

The *pvcs *and *pvmsp3-α *genes were used. Nested PCR and PCR-RFLP were carried out, following the previously described procedure [25,26]. For the *pvcs *gene, five size variants: 650, 680, 700, 720, and 750 base pairs were coded as a, b, c, d, and e, respectively. VK type was labelled as VK210 or VK247 according to the results of PCR-RFLP. For the *pvmsp3-α *gene, three size variants: 1,900, 1,500 and 1,100 base pairs, were labelled A, B, and C, respectively. For PCR-RFLP analysis by *Hha *I, digestion types were labelled as h1-h14 (as sizes A, B, and C: h1-h10, h11-h12, and h13-h14, respectively). Digestion types by *Alu *I were labelled as a1-a24 (as size A, B, and C: a1-a16, a17-a19, and a20-a24, respectively).

Multiplicity of infection (MOI) was estimated by genotyping results in two loci: the *pvcs *gene and the *pvmsp3-α *gene. It was defined as the minimum number of combinations of MOI at each locus. In each gene, MOI was estimated by the number of bands on a PCR product gel picture. In the case of the *pvcs *gene, even if only one band was observed on the PCR product picture, it was estimated that the MOI was two when the results of PCR-RFLP showed both VK types: VK210 and VK247.

### Statistical analysis

The baseline characteristics of the patients in the SAT and DOT groups were compared using chi-square, Student's t, and Mann-Whitney *U *tests, depending on the type and distribution of the data. Age was categorized into three groups, 3-7 years, 8-13 years, and ≥14 years, according to the age-adjusted primaquine regimen. The cut-off level of the total primaquine dose per body weight (2.75 mg/kg) was used to indicate inadequate dose (< 2.75 mg/kg), as reported in a previous study [14]. The incidence rates of *P. vivax *reappearance observed among patients in the SAT and DOT groups were calculated and compared. Cox's proportional hazard regression modelling was used to examine factors related to the first reappearance of *P. vivax *malaria parasitaemia. The analysis was performed using Stata 8.0 computer package (StataCorp., USA).

## Results

### Enrolment and baseline characteristics of the patients

A total of 216 patients were enrolled between 26 May 2007 and 31 October 2008 and randomized into either the SAT group (107 patients) or the DOT group (109 patients). Over half of the patients (n = 123; 57%) were enrolled into the study during the high malaria transmission season in the study area (April to June). G6PD mutations were found in 22% of the patients (homozygous, 2%; hemizygous, 9%; heterozygous, 11%). All mutations were G6PD Mahidol. Despite the random allocation, patients in the DOT group were younger than those in the SAT group (Mann-Whitney *U *test: *p *= 0.004 for continuous age variable, chi-square test: *p = *0.001 for age groups); other baseline characteristics were comparable between DOT and SAT groups (Table 1).

**Table 1:** Baseline characteristics, by treatment group (N = 216)

Variables	DOT	SAT
**Number of patients**	n = 109	n = 107
**Female; N (%)**	45 (41)	41 (38)
**Age group; N (%)**		
3-7 years	34 (31)	20 (19)
8-13 years	26 (24)	13 (12)
≥ 14 years	49 (45)	74 (69)
**G6PD mutation status; N (%)**		
Wild-type	81 (75)	84 (81)
G6PD Mahidol: Homozygous	2 (2)	2 (2)
G6PD Mahidol: Hemizygous	9 (8)	11 (10)
G6PD Mahidol: Heterozygous	16 (15)	7 (7)
**Body temperature **at Day 0 (°C); Mean ± SD	37.8 ± 1.2	37.8 ± 1.2
**Duration of fever pre-treatment; N (%)**		
0-2 days	62 (57)	50 (47)
3+ days	46 (43)	57 (53)
**Parasite count **at Day 0 (/µl); Geometric mean (range)	1,890 (32-75600)	2,533 (32-400000)
**Gametocyte presence **at Day 0; N (%)	45 (41)	37 (35)
**Gametocyte count **at Day 0 (/µl)^; ^Geometric mean (range)	141 (16-1600)	141 (16-8160)
***P. vivax *genotype **(*pvcs *gene) infected at Day 0; N (%)		
VK210	95 (91)	84 (80)
VK247	4 (4)	12 (11)
VK210&VK247	5 (5)	9 (9)
**Number of *P. vivax *genotype infections**; N (%)		
Single	85 (82)	84 (80)
Multiple infection	19 (18)	21 (20)

### Incidence rate of the reappearance of *P. vivax *parasitaemia

Of the 216 patients, 187 (87%) completed their 90-day follow-up period; 90 (83%) patients and 97 (91%) patients were in the DOT and SAT groups, respectively. Reappearance of *P. vivax *parasitaemia during follow-up was observed in 15 patients (8%). The overall incidence rates of *P. vivax *parasitaemia reappearance (per 10,000 person-days) at Days 28, 60, and 90, were 1.67, 4.78, and 8.43, respectively. The median (min-max) time to reappearance of *P. vivax *parasitaemia was 68 (13-90) days.

**Figure 1:**
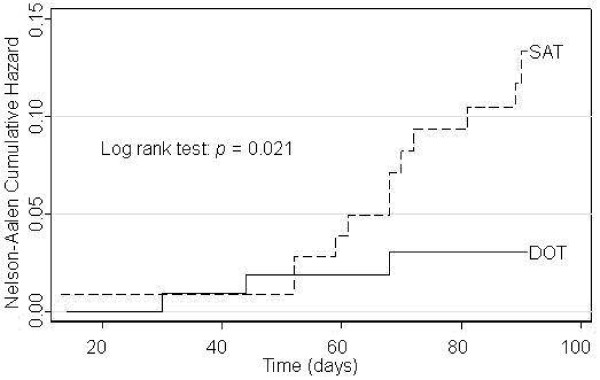
**Nelson-Aalen cumulative hazard curves, by treatment group**.

The rate of *P. vivax *reappearance in the DOT group was significantly lower than the SAT group (Figure 1). The reappearance rates at Days 28, 60, and 90, were 0, 3.18, and 3.37, respectively, in the DOT group, compared with 3.35, 6.39, and 13.49 in the SAT group.

### Factors related to the reappearance of *P. vivax *malaria parasitaemia

In the univariate analysis, treatment group, total primaquine dose per bodyweight, parasite count on admission, and multiple *P. vivax*-genotype infection were significantly associated with reappearance of *P. vivax *malaria (Table 2). There was a borderline positive association between appearance of *P. falciparum *parasitaemia during the follow-up period and risk of *P. vivax *reappearance (crude HR 4.01, 95% CI 0.90-17.77). G6PD mutation status, infected *P. vivax *genotype (VK type), gender, presence of gametocyte at baseline, and *P. vivax *acquisition month did not show statistically significant associations.

**Table 2:** Crude and adjusted hazard ratio (HR) and 95% confidence interval (CI) for factors related to *P. vivax *reappearance

Variables	Reappearance N (%)	Crude HR (95% CI)	Adjusted HR* (95% CI)
**Treatment group**			
DOT	3 (2.8)	1	1
SAT	12 (11.2)	3.98 (1.12-14.09)	6.21 (1.39-27.79)
**Age group**			
3-7 years	6 (11.1)	1	1
8-13 years	1 (2.6)	0.21 (0.03-1.72)	0.02 (0.0008-0.54)
≥14 years	8 (6.5)	0.60 (0.21-1.72)	0.62 (0.17-2.32)
**Duration of fever pre-treatment**			
0-2 days	11 (9.8)	1	1
3+ days	4 (3.9)	0.38 (0.12-1.21)	0.16 (0.04-0.68)
**Total primaquine dose/body weight**			
≥ 2.75 mg/kg	13 (6.1)	1	1
< 2.75 mg/kg	2 (50.0)	8.39 (1.89-37.22)	10.45 (2.02-54.03)
**Parasite count at Day0**			
< 10000 /µl	8 (4.3)	1	1
≥ 10000 /µl	6 (20.0)	4.87 (1.69-14.04)	5.43 (1.59-18.54)
**Multiplicity of *P. vivax *genotype infection**	8 (4.7)	1	1
Single	6 (15.8)	5.97 (2.07-17.23)	4.81 (1.53-15.16)
Multiple			
***P. falciparum *infection post-treatment for *P. vivax***			
No	13 (6.3)	1	1
Yes	2 (22.2)	4.01 (0.90-17.77)	13.66 (1.36-137.64)
**Gender**			
Male	10 (7.7)	1	-
Female	5 (5.8)	0.78 (0.27-2.28)	
**Presence of gametocyte**			
No	10 (7.5)	1	-
Yes	4 (4.9)	0.63 (0.19-2.01)	
***pvcs *gene genotype**			
VK210	12 (6.7)	1	-
VK247	1 (6.3)	1.10 (0.14-8.49)	
VK210&VK247	1 (7.1)	1.06 (0.14-8.13)	
**G6PD mutation status**			
Wild-type	12 (7.3)	1	-
G6PD Mahidol Homozygote	0	-	
G6PD Mahidol Hemizygote	2 (10.0)	1.33 (0.30-5.93)	
G6PD Mahidol Heterozygote	1 (4.3)	0.62 (0.081-4.78)	

*Adjusted for variables included in the multivariate Cox regression model

The significant variables from the univariate analysis, with two additional variables (age group and number of days with fever before start of treatment), were included in the multivariate model (Table 2). The reappearance rate for *P. vivax *was significantly higher in the SAT group, compared with the DOT group (adjusted HR 6.21, 95% CI 1.39-27.79). Children aged 8-13 years had the lowest risk of *P. vivax *reappearance. Interestingly, the reappearance rate appeared to be about six times lower among patients who received late treatment, i.e. pre-treatment duration of fever ≥3 days, compared with early treatment (adjusted HR 0.16, 95% CI 0.04-0.68). Inadequate total primaquine dose was also associated with reappearance as those who received a total primaquine dose < 2.75 mg/kg were about 10 times more likely to have a subsequent *P. vivax *attack than those who received a total primaquine dose ≥ 2.75 mg/kg. In addition, parasite-related factors including baseline parasite count ≥ 10,000 /µl, multiple *P. vivax*-genotype infection, and *P. falciparum *detected during follow-up were positively associated with risk of *P. vivax *reappearance (Table 2).

### Adherence to 14-day primaquine treatment among the SAT group

Adherence to primaquine treatment was determined by interviewing patients in the SAT group (response rate = 94%) at Days 7 and 14. Fifteen of 101 patients (15%) reported that they have not taken the drug at least once during the 14 days of treatment. Patients who missed a primaquine dose or doses tended to have a higher risk of reappearance of *P. vivax *parasitaemia than those who took a complete14-day course (13% vs. 9%), although this difference was not statistically significant.

Non-adherence during Week 1 of treatment was reported by 6% of patients, whereas 12% of patients reported that they missed at least one dose of primaquine during Week 2. The median number of missed doses was more than four times higher in the Week 2 of treatment than Week 1. Interestingly, patients who received the treatment within two days of an acute attack tended to non-adhere to treatment in Week 2, compared with Week 1 (17% vs. 2%; Fisher's exact test, *p *= 0.030), whereas those who came to the clinic > 2 days after the initial attack did not show any difference in treatment adherence for Weeks 1 or 2 (7% vs. 9%). Non-adherence was more likely to be reported by males and in children aged 8-13 years, although the differences were not statistically significant.

### Comparison of genotype in primary attack and reappearance of parasitaemia

The parasite genotypes in the primary and subsequent attacks for 15 patients who had reappearance of *P. vivax *were analysed. Blood samples from two patients failed to amplify, and three blood samples of either primary or subsequent attacks from three patients were missing. The *P. vivax *genotype patterns of the remaining 10 patients are summarized in Table 3. Three patients had the same *P. vivax *genotypes in both primary attack and reappearance. Five patients showed different genotype patterns in the primary episode and subsequent attack.

**Table 3:** Comparison of *P. vivax *genotypes in primary attack and reappearance(s) (N = 10)

	Case	Primary attack	First reappearance		Second reappearance	
		Parasite count (/µl) *pvcs, pvmsp3α *gene	Day	Parasite count (/µl) *pvcs, pvmsp3α *gene	Day	Parasite count (/µl) *pvcs, pvmsp3α *gene

Same*	**1**	> 400000 c_VK210, A_h8a7	68	160 c_VK210, A_h8a7	91 (159)	800 c_VK210, A_h8a7
	**2**	8800 be_VK210, A_h6a7	30	6640 be_VK210, A_h6a7	29 (59)	7440 Missing data
	**3**	3080 c_VK210, A_h1a2	68	16480 c_VK210, A_h1a2		

**Different^‡^**	**4**	29360 c_VK247, A_hm^†^am^†^	89	400 b_VK210, A_h1a15		
	**5**	30640 b_VK210, A_h1a4	90	22880 b_VK210, A_h4a6		
	**6**	1280 d_VK210&247, A_h2a3	52	112 c_VK210, A_h5a8		
	**7**	7440 ab_VK210, AC^§^	52	12320 b_VK210, A_h7a2		
	**8**	19360 be_VK210, A_h3a1	81	33440 b_VK210, A_h1a4		

**Ambiguous**	**9**	16560 a_VK210, AC^§^	68	2040 c_VK210, C_h1a3		
	**10**	16000 c_VK210, A_hm^†^a2	59	2720 c_VK210, A_h5a2	60 (119)	64 c_VK210, A_h1a2

* For two markers, all of the gel picture patterns in the primary-attack parasite and reappearance parasite were the same.

^† ^Digestion pattern not classified, because sum of PCR-RFLP fragment sizes exceeded uncut gene size.

^‡ ^For two markers, at least one band in the gel picture patterns in the primary attack and in the reappearance parasite was different.

^§ ^Digestion pattern not classified because of multiple-genotype infection classified by PCR result for the *pvmsp3-α *gene.

Among the patients who experienced reappearance of *P. vivax *malaria, the type B *pvmsp3-α *gene was not found in either the parasites responsible for the primary attack nor the reappearance. The proportion of *pvcs *gene genotype, VK type (regardless of gene size), and the *pvmsp3-α *gene size of the *P. vivax *parasite in the primary attack and in the reappearance of parasitaemia did not reveal any significant difference.

## Discussion

Adherence to a 14-day primaquine regimen for the radical treatment of *P. vivax *hypnozoites, especially after symptoms have subsided, is a serious concern. In this study we found the non-adherence rate in treatment Week 2 was double compared to Week 1. The 15% non-adherence rate is probably an underestimate since some patients may not have reported missing a dose. Moreover, the home visit on Day 7 among the SAT group may increase the adherence rate among patients in that group, which further underestimates the reported non-adherence rate, compared with the real situation. The DOT method ensures that patients complete the full course of treatment. The findings of this study suggest that, compared with self-administration, DOT increases the likelihood of radical cure in *P. vivax *malaria infection. Patients with supervised therapy were about six times less likely to have *P. vivax *reappearance within the 90-day follow-up period. The protective effect of the DOT is likely to be larger than the estimate, if the underestimation of non-adherence rate in the SAT group was taken into account.

This study also showed that total primaquine dose per body weight < 2.75 mg/kg was associated with reappearance of *P. vivax *parasitaemia. This indicates that an inadequate dose of primaquine during the primary attack may increase the risk of a subsequent *P. vivax *attack. In practice, weight-independent doses are used because of the difficulties of using weight-adjusted doses in the field. Therefore, total primaquine dose per body weight varies considerably; e.g., patients who weigh 60 kg and 80 kg receive 3.5 mg/kg and 2.6 mg/kg, respectively, adhering to the adult regimen of "15 mg of primaquine daily for 14 days". Treatment failure due to an inadequate dose may be misinterpreted as drug resistance. This misinterpretation may occur not only between individuals, but also between areas because average body weight differs between countries and ethnic groups. The average bodyweight of patients in this study aged ≥ 14 years was 52.7 kg compared to 67.3 kg in a study in Brazil [14]. Therefore, weight-adjusted doses should be considered when assessing whether primaquine-resistant *P. vivax *is present. In addition, weight-based dosing schedules for primaquine should be more appropriate than the existing nationwide age-adjusted guideline for primaquine treatment due to a wide range of average body weight among different populations.

Poor adherence was more likely to be reported by males or children aged 8-13 years. While children aged 8-13 years showed the highest rate of non-adherence to primaquine treatment, they had the lowest rate of reappearance of *P. vivax *parasitaemia. This may be explained by the relatively high dose of primaquine that children aged 8-13 years received compared with the other age groups (age group 3-7 years: 4.74 ± 0.95 mg/kg, 8-13 years: 5.07 ± 0.95 mg/kg, ≥ 14 years: 4.19 ± 0.98 mg/kg). Even if these children skipped a dose once or twice, the total primaquine dose would still be sufficient to constitute a "therapeutic dose".

Higher multiple *P. vivax*-genotype infections were associated with increased risk of the reappearance of *P. vivax *parasitaemia, which may be the result of acquired genotype-specific host immunity [27]. Patients who suffer from mixed genotype infections may develop genotype-specific acquired immunity to only the dominant genotype in the primary attack. As a consequence, the low-level genotype in the primary attack could reappear more easily than the dominant genotype which may be suppressed by acquired genotype-specific immunity. In contrast, patients with a single genotype infection, whose acquired immunity develops during primary infection, can suppress the reappearing parasites that are caused by hypnozoites with the same genotype as those in the primary attack. In this study, at least six mixed-genotype infections were found among patients who had *P. vivax *reappearance; however, the conventional PCR used to analyse the genotype in this study was unable to identify the dominant genotype within an individual host. Using real-time PCR to determine the proportion of each genotype in the primary attack may help understand whether the dominant or the low-level genotype actually reappears.

The risk of the reappearance of *P. vivax *in this study was related to the level of parasitaemia on admission, consistent with a previous study from Brazil [14]. In Thailand, the major strain of the *P. vivax *parasite is the Chesson strain, which can produce about equal numbers of hepatic schizonts and hypnozoites [28]. This ratio remains constant regardless of the initial number of sporozoites inoculated [29]. Therefore, the absolute number of hypnozoites should increase with parasite count, which subsequently increases the probability of relapse.

The duration of fever before initial treatment was significantly associated with the reappearance of *P. vivax*. Patients who received early treatment (≤ 2 days) were more likely to develop a repeat *P. vivax *attack. This finding is possibly explained by the relationship between the timing of primaquine administration and the growth and stabilization of the hypnozoites. Research has not yet confirmed exactly how primaquine works on hypnozoites. However, primaquine is thought to affect the parasite mitochondrial electron transport chain [30]. Because hypnozoites are "dormant", they cannot replace their damaged mitochondria and become extinct [31]. The hypnozoites can be recognized three days after the host has been infected with *P. vivax*, and the size of hypnozoites becomes stable 7-15 days after infection [32-34]. However, it is unclear when the hypnozoites' function becomes "dormant". Since the incubation period of *P. vivax *is 12-17 days, some hypnozoites may not have stabilized yet during the first few days after the acute attack. Therefore, if primaquine administration is started earlier after an initial attack, the drug's effects may be less than expected because the hypnozoites are not "dormant" enough. The association between relapse rate and timing of treatment may be explained by the difference in the non-adherence rate between patients treated early and those who had treatment delay. In this study, non-adherence was found in 13% of patients who received late treatment, as compared with 17% of those with early treatment. However, this difference was not statistically significant.

The appearance of *P. falciparum *parasitaemia during the follow-up period was found to be a risk factor. However, the sample size was too small to obtain reliable results. Further research is needed to confirm these findings.

The *pvcs *and *pvmsp3-α *gene loci were used to compare the parasite genotypes in the primary attack and the reappearance of parasitaemia. The results showed a similar genotype pattern in the primary attack and the reappearance of parasitaemia. No particular genotypes were found to be associated with reappearance. Recently, *P. vivax *genome sequencing has revealed some genes that are likely related to dormancy or differentiation [35], and may be associated with risk of relapse. However, future research using these genes is needed to explore the association. In terms of comparison of genotypes between primary and subsequent attacks, most of the genotypes observed in the parasites in a primary attack were not exactly the same as the reappearance parasites. The genotypic difference in the reappearing parasite cannot distinguish relapse from heterozygous hypnozoite or re-infection [27]. Since the episodes of reappearance were observed within 90 days of the initial attack, when the risk of relapse was highest in this low malaria-transmission area, the parasitaemia reappearances observed were most likely due to relapse.

## Conclusions

This study supports the supposition that poor adherence to 14-day primaquine treatment can affect the effectiveness of radical therapy for *P. vivax *malaria infection; DOT should be considered for improving compliance to 14-day primaquine treatment, since it reduces the risk of relapse. In areas where both tuberculosis and vivax malaria are prevalent, integrating DOT for *P. vivax *with existing DOT system for treatment of tuberculosis, where health workers had been trained for house visit to deliver drugs, should have a major impact on reducing disease transmission and on the financial burden of these two diseases. Inadequate dose of primaquine is also one of the most important factors for radical cure of *P. vivax*; weight-adjusted doses should be considered and the most practical way to apply weight-adjusted doses should be sought.

## Competing interests

The authors declare that they have no competing interests.

## Authors' contributions

RT, MI, JKo, JKa, SPuk and PS designed the study protocol; SPua, NT, and WM collected the data; RT and MI conducted the PCR analysis; RT, JKa and PS analysed and interpreted the data; RT, SL, and NJD drafted the manuscript. All authors read and approved the final manuscript.
